# Original Fluorescent Ligand-Based Assays Open New Perspectives in G-Protein Coupled Receptor Drug Screening

**DOI:** 10.3390/ph4010202

**Published:** 2011-01-18

**Authors:** Martin Cottet, Orestis Faklaris, Jurriaan M. Zwier, Eric Trinquet, Jean-Philippe Pin, Thierry Durroux

**Affiliations:** 1 Institut de Génomique Fonctionnelle, CNRS, UMR 5203, Montpellier, France and INSERM, U. 661, Montpellier, France and Université Montpellier,1,2, Montpellier, France; 2 CIS bio international, BP 84175, F-30204 Bagnols sur Cèze Cedex, France

**Keywords:** screening assay, fluorescence, lanthanides, time resolved FRET, GPCR

## Abstract

The identification of new drugs exhibiting reduced adverse side-effects constitutes a great challenge for the next decade. Various steps are needed to screen for good ligand candidates and one of them is the evaluation of their binding properties. New strategies based on fluorescence measurement constitute excellent alternatives to the traditional radioactive assays. Less hazardous, faster and cheaper, these methods also exhibit very good sensitivity and can be used on various biological models such as heterologous expression systems or native tissues.

## 1. Introduction

G-protein coupled receptors (GPCRs) are receptors that constitute a large protein family present throughout the organism and covering a majority of vital functions. These receptors, which share a common structural backbone composed of seven transmembrane domains, respond to diverse stimuli ranging from a single photon activating the rhodopsin receptor to large proteins binding and activating receptors such as luteinizing hormone receptor. The importance of this family of receptors is mirrored by the pharmaceutical research interest in them, with over 30% of drugs being directed against these targets [1].

Moreover, it has been shown during the past two decades that receptors can couple to more than one signaling pathway and that some ligands, named biased agonists, have the ability to stimulate selectively only one of them [2]. The emergence of this new concept is crucial in medicinal pharmacology since side-effects of some drugs have been linked to the activation of secondary signaling pathways. The possibility to screen for biased agonists, which only activate the principal signaling pathway and therefore limit adverse side-effects, has renewed the interest in screening GPCR ligands. Furthermore, since the coupling properties of a GPCR can also be dependent on whether the GPCR is engaged in a homo- or a hetero-oligomer [3,4], the identification and the characterization of dimer complexes as potential new therapeutic targets are thus of importance.

In this article we review the development of different methods based on fluorescent ligands as tracers to perform efficient ligand binding assays on GPCRs. Indeed, the synthesis of high affinity fluorescent ligands for various classes of GPCRs and the emergence of new concepts on GPCR activation have led to the development of various assays. The sensitivity of these methods is related to the biochemical properties of the fluorescent ligands and whether the receptors are expressed in a native context or in a heterologous expression system.

## 2. Radioactive Binding Assays

**Figure 1:**
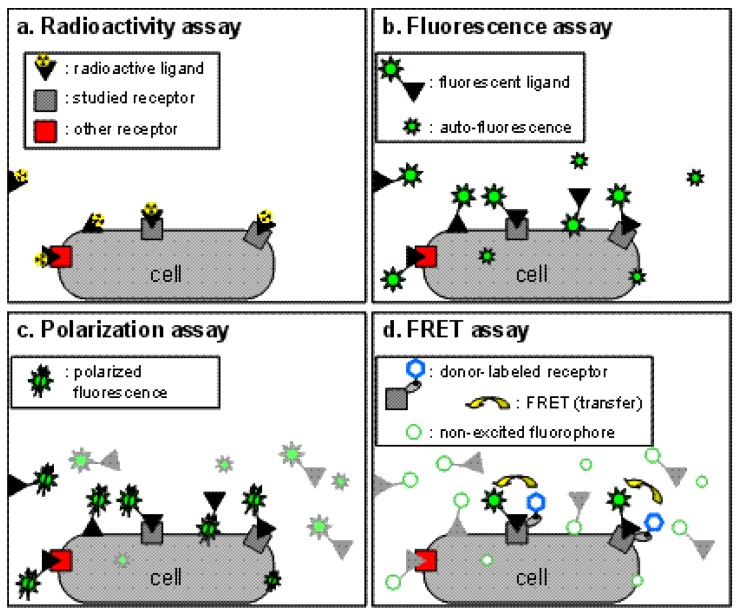
Different binding assay methods. (a) Radioactive binding assay performed in a heterogeneous format, with washing steps to eliminate the excess of unbound radioactive ligand. Noise can come from non-specifically bound ligand;(b) Fluorescence binding assay performed similarly to radioactive assays. Noise can result from auto-fluorescence of the sample and non-specifically bound ligands; (c) Polarization binding assay performed in a homogeneous format: the excess of labeled ligand is not removed as only the immobilized fraction (e.g. bound ligands) will retain the fluorescence polarization; (d) Homogeneous FRET-based binding assay, with only the specifically bound fluorescent ligand participating in the FRET, therefore increasing the signal-to-noise ratio.

Binding assays are the cornerstone of all pharmacological studies and are indispensable for the development of new drugs targeting specific receptors. Traditionally, assays based on radioactive probes (usually tritiated or iodinated ligands) have been used successfully to determine ligand binding characteristics because of the high sensitivity of the techniques and the possibility to perform experiments on unmodified receptors expressed in native tissues and in transfected cells (Figure 1a). However, the use of radioactive ligands as tracers in binding assays presents several drawbacks, both technical and financial. Technically, a classic radioactive binding assay cannot be performed in a homogeneous format. Consequently the assay requires multiple washing steps before reading the radioactivity. This adds a complexity to the procedure and thus makes these assays more difficult to perform in high-throughput screening and leads to an extra cost. Additionally, the washing steps prevent any possibility of carrying out kinetics experiments on a single sample. Over the past decade, strategies such as scintillation proximity assays (SPA) which can be performed in homogeneous conditions have been developed, but are still expensive due to the production cost of beads. A second technical drawback stems from the nature of the radioactive probe with which it is difficult, in part for health reasons, to perform saturation assays which necessitate high concentrations of radioactive ligand. Furthermore, due to the hazardous nature of the compounds, the use of radioactive probes has some restrictions in term of radioactive waste disposal, delimitation of working area and staff medical follow-up. As a result, and because of high costs resulting from these restrictions, other techniques have been introduced to replace the use of radioactive binding assays, such as fluorescence techniques, without completely supplanting them.

## 3. Fluorescent Ligands to Perform Binding Assays

Fluorescence-based binding assays are promising alternatives to radioactive assays for several reasons. Firstly, the fluorescent probes needed for such tests are safe to use and they do not present the same restrictions of radioactive probes, resulting in easier to implement assays. Secondly, bright and stable probes have been developed over the past two decades and are now commercially available. Thirdly, because of the structure/activity relationship analyses, potent fluorescent ligands have been designed and synthesized for numerous receptors. Indeed, the addition of a fluorescent probe to a ligand is not as “harmless” as the tritiation of the ligand. It adds steric hindrance as well as hydrophobicity modifications which can significantly alter the pharmacological properties of the ligand, sometimes diminishing its affinity to such an extent that it can become useless in pharmacological studies.

Finally, instruments needed to measure fluorescence are also becoming more sensitive and are very widespread as they are polyvalent and frequently used to perform common fluorescence or luminescence-based assays. Various experiment formats such as fluorescent intensity measurement, fluorescence polarization and energy transfer between probes can be used to evaluate ligand binding properties and they deeply impact the sensitivity of the test.

## 4. Direct Measurement of Fluorescent Intensity of Ligand Bound onto the Receptor

This approach is probably the simplest one to perform and consists in the measurement of the fluorescent ligand bound fraction after separation from the free fraction. It therefore cannot be performed under homogeneous conditions. The sensitivity of this method is often questionable because of the auto-fluorescence of biological preparations. This issue can be addressed by temporally selecting the fluorescence of interest with strategies such as DELFIA [5]. These approaches are based on the use of lanthanide fluorophores exhibiting a long fluorescence lifetime which persists after the extinction of the short-lived auto-fluorescence (see details in time-resolved FRET section). A second factor which can decrease the signal-to-noise ratio is the non-specific binding of the fluorescent ligand. Although the same drawback exists for radioactive assays, the hydrophobicity of the fluorophore linked to the ligand can increase its non-specific binding (Figure 1b). Taken together, these drawbacks make it difficult to directly implement fluorescence assays to study pharmacological properties.

## 5. Fluorescence Polarization Assays

Fluorescence polarization techniques can be used to measure more precisely the amount of bound fluorescent ligand. These techniques are based on the excitation of the biological sample with polarized light. Constrained fluorophores will emit highly polarized fluorescence. On the other hand, freely moving fluorophores which can easily rotate during the short period between their excitation and the emission of fluorescence will scramble this polarization. Therefore, when exciting the sample with a polarized light, a fluorescent ligand freely diffusing in the medium with an important molecular mobility will emit a non polarized fluorescence, whereas upon binding a receptor, the molecular mobility of the fluorophore within the receptor-fluorescent ligand complex will frequently decrease, and a polarized fluorescence emission will be observed (Figure 1c), leading to a high fluorescence anisotropy.

Fluorescence polarization assays, like the ones previously mentioned, can be performed on receptors expressed either in native tissues or in transfected cells. Moreover, these assays can be performed in homogeneous conditions: fluorescent ligands are simply added to the biological sample and no washing steps are required to measure fluorescence polarization, making it very simple to adapt for high-throughput screening and allowing straightforward kinetics studies [6]. However, the affinity of the fluorescent ligands should be high enough to bind to the receptor at low concentrations and therefore get a bound ligand-to-free ligand ratio high enough to give a significant fluorescent polarization signal.

Although the measures are very reproducible, the measurement window is very narrow, the polarization not being total or completely inexistent due to contamination by non-specific fluorescence and auto-fluorescence. Such a narrow window can be challenging when performing high-throughput screening as the variation between positive and negative results can be too small to guarantee simple interpretation and can lead to false negatives. This probably explains why fluorescent polarization assays which have been described as fast, sensitive and inexpensive [7] have not been extensively used [8-12].

## 6. FRET-Based Binding Assays

As mentioned above, the sensitivity of a binding assay is highly dependent on the non-specific binding which will decrease the signal-to-noise ratio and therefore the size of the measurement window, leading to less reliable and reproducible end results. Furthermore, a lack of selectivity (and not of affinity) of a ligand can be detrimental to evaluate the affinity of a ligand for the receptor of interest. Indeed, radioactive and fluorescence intensity or fluorescence polarization based assays cannot discriminate between the specific binding of a ligand on two different receptors.

**Figure 2:**
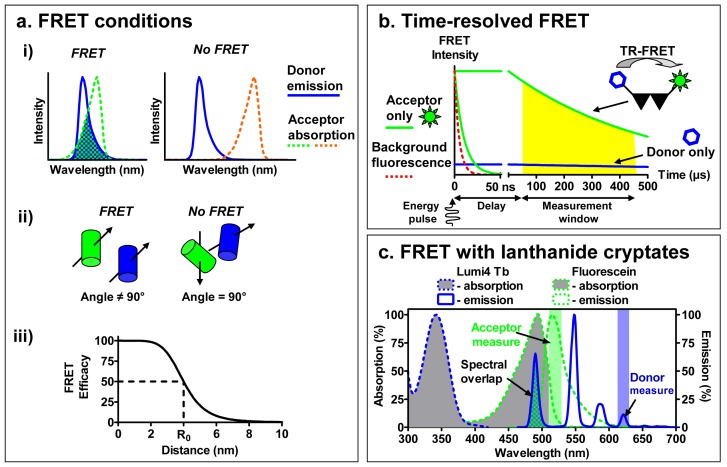
Principles of FRET and TR-FRET. (a) Three conditions are needed for Fluorescence Resonance Energy Transfer (FRET): i) energy compatibility, *i.e.* overlapping of the donor emission and the acceptor excitation; ii) alignment of the fluorophores' dipoles and iii) proximity of the fluorophores, the FRET efficacy rapidly decreases with the distance between the fluorophores; (b) Time-resolved FRET (TR-FRET) is based on the use of fluorophores with long fluorescence lifetimes such as lanthanide cryptates. By briefly exciting the sample and imposing a delay before measuring the fluorescence, all classic fluorescence phenomena disappear leaving only the signal of the donor or the acceptor engaged in the FRET, depending on the selected wavelength; (c) Lanthanide cryptates with europium (Eu-PBBP) or terbium (Lumi4-Tb) present numerous and well resolved emission wavelengths when excited in the UV (300-350 nm), making them ideal donor fluorophores capable of transferring on either green fluorescein-like or near infra-red fluorophores.

To overcome this challenge, binding assays based on fluorescence resonance energy transfer (FRET) have been developed (Figure 1d). Briefly, FRET occurs between a ‘donor’ fluorophore and an ‘acceptor’ fluorophore, which can be carried either by the ligand or by the receptor. Three parameters condition FRET efficacy [13] (Figure 2a): (i) the two fluorophores have to exhibit energy compatibility; (ii) the dipole moments of the fluorophores have to be correctly oriented, the FRET being null when dipoles are perpendicular and maximum when they are parallel; and (iii) the fluorophores have to be close enough, usually less than 10 nm apart.

Among these parameters, the energy compatibility is probably the most crucial to perform FRET between a receptor and a ligand. The emission spectrum of the ‘donor’ should overlap the excitation spectrum of the ‘acceptor’ and the extent of overlap directly impacts the FRET efficacy. However looking for a large overlap to perform FRET is often associated with a poor separation between the excitation spectra of donor and acceptor and between their emission spectra. For the end user this leads to extensive calculations in order to differentiate the acceptor fluorophore engaged in a FRET from an acceptor directly excited and the bleed-through fluorescence of the excited donor.

**Figure 3:**
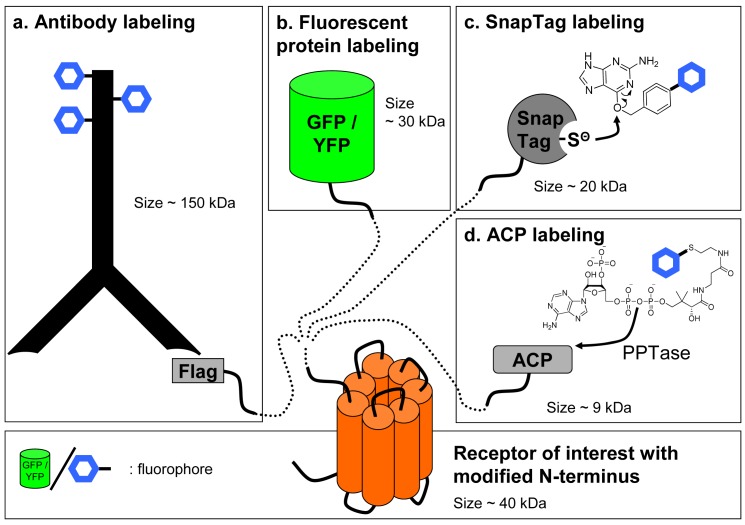
Labeling techniques for G-protein coupled receptors modified at their N-terminus. (a) Labeling with an antibody directed against an epitope; (b) Fusion of a fluorescent protein to the receptor; (c) SnapTag labeling of the receptor by fusion of a SnapTag suicide enzyme and addition of a fluorescently-labeled substrate (benzylguanine); (d) ACP labeling by fusion of an ACP epitope to which binds part of fluorescent coenzyme-A catalyzed by PPTase.

In contrast with ligands that are usually labeled by chemical approaches, labeling of the receptor can be achieved by various strategies. Non-covalent labeling can be performed with antibodies directed against the receptor to label it specifically. Although this approach is convenient for labelling receptors in a native context, obtaining high affinity and highly selective antibodies is difficult especially against subtypes of receptors of the same family. Consequently, it has been deemed easier to fuse an artificial tag at the N-terminal, extra-cellular end, of the GPCR and to label the receptor with an antibody directed against this tag (Figure 3a).

An alternative to the use of reversible antibody labeling of chimeric receptors can be the direct labeling of the receptor by fusing to its N-terminal extremity a fluorescent protein using molecular engineering, traditionally with proteins derived from the Green Fluorescent Protein (GFP) [14] (Figure 3b). This strategy has been successfully used with GFP or YFP fused to the receptor as the donor and ligands linked to lissamine or Bodipy [15,16] or a non fluorescent quencher [17] acting as the acceptor. However, these labeling methods suffer from the common drawbacks associated with FRET and fluorescence assays. Firstly, although various fluorescent proteins derived from GFP have been genetically engineered to present fluorescent spectra ranging from blue to deep red, these proteins generally exhibit large excitation and emission spectra making it sometimes difficult to excite only the donor or to detect only the acceptor emission. Therefore, to achieve a better separation, excitation of the donor and emission of the acceptor are not performed at their maximum, leading to a decrease in the signal amplitude. Secondly, the signal-to-noise ratio is also dramatically decreased because of the medium and biological sample's auto-fluorescence as well as the intracellular store of fluorescent receptors which have either internalized or have not been targeted to the cell membrane.

## 7. Time Resolved FRET with Lanthanide Cryptates to Increase Signal to Noise Ratio

To increase the sensitivity of FRET approaches, a new type of FRET was developed to address these issues implementing lanthanides as donor fluorophores [18]. Whereas classical fluorophores have fluorescence lifetimes in the range of a few nanoseconds, lanthanides are characterized by much longer fluorescence lifetimes, between several hundred microseconds to a few milliseconds. This distinctiveness has led to the development of Time-Resolved FRET (TR-FRET), a technique in which the donor fluorophore is excited with a laser or a flash-lamp before measuring the emission signal. The imposed time-delay results in a selective temporal filtering of the cell auto-fluorescence signal and other shortlived fluorescence, characterized by fluorescence lifetimes of several nanoseconds. The acquisition of the signal coming from the longer fluorescence lifetimes of lanthanides after the imposed time-delay drastically reduces the background noise and consequently improves the signal-to-noise ratio [19].

A second advantage of the use of lanthanides is that they are excited in the near-UV and have an important Stokes shift, making possible the selective excitation of only the donor and subsequent separate measurement of the donor or acceptor fluorescence (Figure 2c). The excitation and emission spectra can be modified by the cage and sensitizer molecule to which the lanthanides are complexed. When lanthanide-ions are complexed with either a chelate or a cryptate, they are partly protected from the quenching by water molecules, and they can be linked to the protein of interest. By contrast with chelates whose interactions with lanthanide are sensitive to some divalent ions (Mn^2+^, Mg^2+^ and Ca^2+^), the interactions of lanthanide with cryptates are very stable leading to an easy interpretation of the data. Cryptates of europium generally exhibit an excitation peak between 300 and 350 nm and emission peaks around 585, 605, 620 and 700 nm. Due to the narrow bandwidth of the donor emission, near infra-red acceptors emitting around 665 nm can be used to selectively detect the delayed acceptor emission, with a minimum background from the donor emission. More recently, a new lanthanide cryptate using terbium instead of europium, Lumi4-Tb, has been developed and exhibits a brighter fluorescence. Moreover, due to its emission bands at 485 nm and 620 nm, it can be used to perform FRET experiments in association with both green or near infra-red emitting fluorophores. As a result, the increase in signal-to-noise ratio combined with the distinct separation of the donor and acceptor spectra render the use of TR-FRET very interesting for performing receptor binding studies with fluorescent ligands bound to a receptor labeled with a lanthanide cryptate.

Labeling a receptor with lanthanide cryptates cannot be achieved by molecular engineering. Two methods can be currently used. The first labeling method implies the labeling of purified antibodies with lanthanide cryptates by biochemical approaches [20]. If a high affinity antibody is available (typically in the nanomolar range), the concentration of labeled antibody added can be minimal and the FRET assays can then be performed in a homogeneous format, eliminating all final washing steps [12]. Although sensitive, this strategy presents two main drawbacks. Firstly, because antibodies are large molecules (around 150 kDa) and represent almost three times the size of the receptor studied, they induce a steric hindrance when bound onto the receptor which can impact the binding properties of the receptor for the ligands. Secondly, although kinetics experiments are feasible, the FRET signal will depend on two parameters: the binding kinetic of the ligand and the labeling kinetic of the receptor by the antibodies.

Such drawbacks have pushed the development of new techniques for a covalent receptor labeling with a tag smaller than antibodies and which can easily be derivatized with lanthanide cryptates. Labeling with a suicide enzyme can fulfill these criteria. The principle is to fuse a suicide enzyme to the receptor by molecular engineering. The fusion to the N-terminus is interesting since the enzyme fused to the GPCR becomes accessible to the substrate when receptors are targeted to the cell surface. Such techniques have recently been developed by Johnsson and colleagues [21-25]. Briefly, a 20 kDa suicide enzyme (SnapTag) derived from the DNA repair enzyme alkylguanine transferase (AGT) is fused to the N-terminus of the receptor. This enzyme has a reactive cysteine which covalently binds to the oxygen of a benzylguanine substrate. Providing a substrate carrying the fluorophore of choice will lead to the fluorescent labeling of the SnapTagged receptor (Figure 3c).

**Figure 4:**
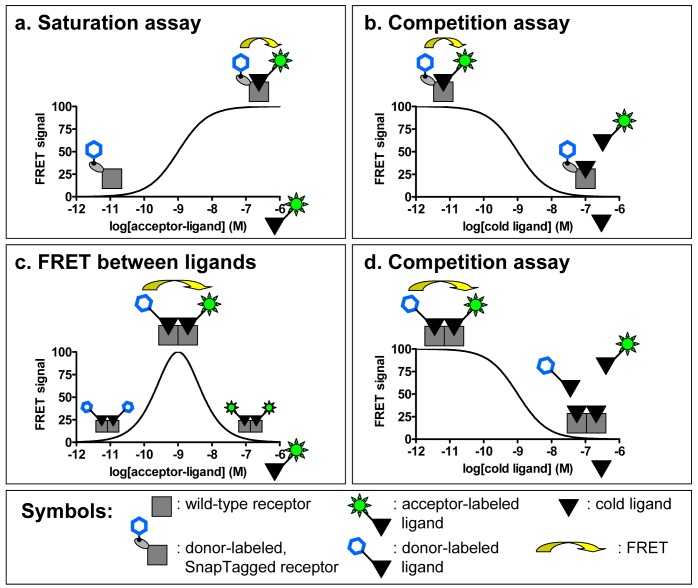
Principle of FRET-based binding assays. (a) Saturation assays can be performed simply in homogeneous conditions with a donor-labeled receptor (e.g. by fusing the receptor to a SnapTag suicide enzyme). FRET signal corresponds to the specifically bound fluorescent ligand; (b) The bound fluorescent ligand can be displaced by increasing concentrations of unlabeled “cold” ligand; (c) FRET can also be measured in optimum conditions when a donor-labeled ligand and an acceptor-labeled one bind simultaneously to a GPCR oligomer; (d) This specific FRET can be displaced by increasing the concentrations of unlabeled ligand, providing the basis for a possible high-throughput screening technique.

This technique has the advantage of allowing a complete and permanent labeling of surface receptors, maximizing the FRET signal between the receptor and the fluorescent ligand [26]. Moreover, fusion of SnapTag to the N-terminus of the receptor does not alter its pharmacological properties as has been shown for numerous receptors [26,27]. Because the SnapTag substrate can be derived with any fluorophore, this technique is also very interesting for performing TR-FRET binding assays with a wide range of commercially available or easily producible fluorescent ligands (Figure 4a, b). This strategy has been used with great success on a large panel of GPCRs (class A receptors from bioamine to peptide receptors, class B and class C receptors) [27]. Interestingly, kinetics experiments can be performed to determine the dissociation and association constant of a ligand for a receptor since the FRET signal depends only on the binding of the ligand.

Similar approaches have recently been developed: the covalent labeling of a protein can be performed using Acyl-Carrier protein (ACP) inserted in the sequence of the receptor and a fluorophore-conjugated phosphopantetheinyl moiety of coenzyme-A. This reaction is catalyzed by phosphopantetheinyl transferase (PPTase) added to the medium [28] (Figure 3d). This method was recently used with GPCRs [29,30]. However, without the production of knock-in mice expressing chimeric receptors, all these techniques will be limited to studies in heterologous expression systems without offering a credible alternative to radioactive assays in native tissues.

## 8. Time Resolved FRET Assays in Native Tissues

An original strategy has been developed to perform TR-FRET experiments on receptors expressed in native tissues by taking advantage of the quaternary structure of GPCR complexes. Indeed it has been proposed that receptors can interact with each other and form homo- or hetero-oligomers. Considering the homo-oligomer model for which evidence have been provided both in heterologous expression systems and in native tissues [31], it would be possible to perform FRET with two ligands labeled either with a “donor” fluorophore (europium or terbium cryptates) or an “acceptor” (fluorescein like or near infra-red fluorophores). The simultaneous binding of the two ligands on one receptor oligomer should lead to a FRET signal. The proof of concept was brought on the oxytocin receptor expressed in a heterologous expression system (Cos7 cell line) and in a native tissue, the mammary gland of lactating rat. Binding studies allow us to determine the concentration range for which the fluorescent ligands will label a maximum of surface receptors and the optimal condition to measure FRET between two ligands bound to a GPCR oligomer (Figure 4c). By using high affinity fluorescent ligands and therefore limiting the concentrations of fluorophores needed to label a majority of receptors, the binding can be performed in a homogeneous format, simplifying the assay.

Addition of competing unlabeled oxytocin induces a progressive decrease of the FRET signal which follows a sigmoidal curve (Figure 4d). The IC_50_ obtained by competing the binding of the fluorescent ligands with oxytocin correlates very well with its known affinity for its receptor indicating the specificity of the FRET signal [31]. The FRET measured between ligands could lead to the development of a new type of binding assay. As the resulting FRET is based on the equilibrium of binding of several compounds (donor, acceptor and competitor ligands), this assay would not permit the determination of precise dissociation or kinetic constants. However the homogeneous format of this assay makes it interesting for high-throughput screening.

This method is based on the concept of GPCR oligomerization, and its sensitivity depends directly on the number of receptor oligomers present at the cell surface, as well as the availability of high affinity fluorescent ligands. Nevertheless, similar results have been obtained with different sets of fluorescent ligands on other class A GPCRs such as the vasopressin V1a, V_2_ and dopamine D_2_ receptors expressed in heterologous expression system (Cos7 or CHO cell lines) [31] proving that the concept of this binding assay could be enlarged to other GPCR models. However, the possibility to implement this assay on other native tissues still remains to be verified.

## 9. Conclusions

Development of fluorescence-based assays that propose a credible alternative to highly sensitive radioactivity-based assays constitutes a challenge. Among fluorescence-based tests, TR-FRET strategies represent original solutions since they yield a high signal-to-noise ratio. As previously mentioned, the high sensitivity is partly due to the reduction of short-lived fluorescence but also to the decrease of the non-specific binding signal because of the two levels of specificity. Indeed, only signals provided by fluorescent ligands in proximity of the labeled receptor are measured, reducing the importance of non-specific binding.

In fact, fluorescent ligands reveal themselves to be extremely efficient tools when coupled with TR-FRET technology for performing binding assays and therefore may replace radioactive assays. These recently developed techniques have proven to be particularly robust and reproducible, allowing for a simple adaptation to high-throughput screening procedures (Table 1). These assays benefit from the development of specific fluorescent ligands and the use of lanthanide cryptates which provide an increased signal-to-noise ratio as well as a simplified measurement procedure.

Although fluorescent ligands can evidently bring information through binding studies, their remarkable ability to specifically label receptors can also be a significant advantage for the study of GPCR dynamics, in heterologous expression systems such as cell lines as well as in native tissues. Although these fields require further development than the simple application of ligands to binding assays, information regarding the organization and the functioning of GPCRs in their native environment could prove to be the most important gain acquired with these fluorescent compounds.

## Figures and Tables

**Table 1:** Advantages and drawbacks of different binding assays presented in this article.

			**Advantages**	**Drawbacks**
**Fluorescence**		Radioactivity	• High sensitivity (especially with iodinated ligands)	• Hazardous material
				• Non homogeneous assay
		Intensity	• Simple to implement	• Low signal-to-noise ratio (except in DELPHIA strategy)
		Polarization	• Simple to implement	• Low signal-to-noise ratio (especially with hydrophobic ligands)
			• Homogeneous assay	
	**TR-FRET (between A / B)**	Antibody-labeled receptor / ligand	• Homogeneous assay	• Not relevant to measure ligand binding kinetics
			• High signal-to-noise ratio	
		Covalently labeled receptor / ligand	• Homogeneous assay	• Not relevant to perform binding on native receptors
			• High signal-to-noise ratio	
		Ligand / ligand	• Homogeneous assay	• Relevant only if receptors oligomerize
			• High signal-to-noise ratio	
